# Evaluating the Impact of Hydrophobic Silicon Dioxide in the Interfacial Properties of Lung Surfactant Films

**DOI:** 10.1021/acs.est.1c06885

**Published:** 2022-01-26

**Authors:** Eduardo Guzmán, Eva Santini, Michele Ferrari, Libero Liggieri, Francesca Ravera

**Affiliations:** † Departamento de Química Física, Facultad de Ciencias Químicas, Universidad Complutense de Madrid, Ciudad Universitaria s/n, 28040-Madrid, Spain; ‡ Instituto Pluridisciplinar, Universidad Complutense de Madrid, Paseo de Juan XXIII 1, 28040 Madrid, Spain; § Istituto di Chimica della Materia Condensata e di Tecnologia per l’Energia, UOS Genova-Consiglio Nazionale delle Ricerche (ICMATE-CNR), Via De Marini 6, 16149 Genova, Italy

**Keywords:** lung surfactant, monolayers, rheology, surface pressure, particles, physiological response, pollution

## Abstract

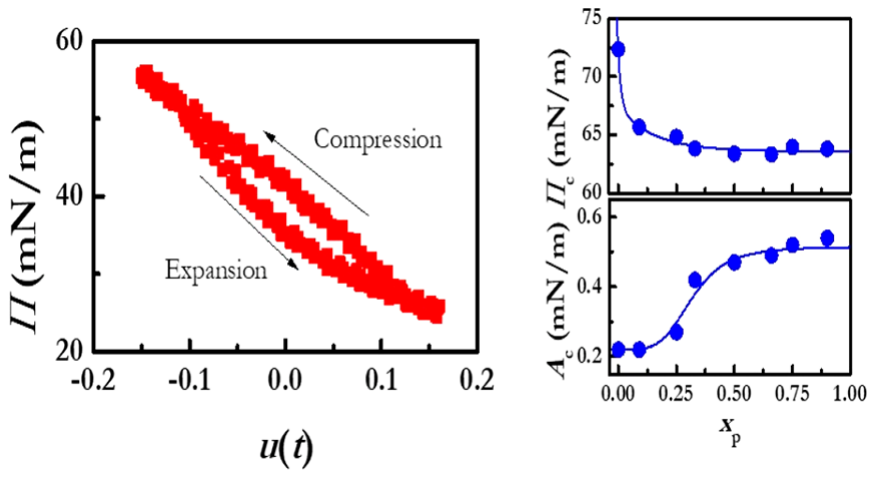

The interaction of hydrophobic silicon dioxide particles (fumed silicon dioxide), as model air pollutants, and Langmuir monolayers of a porcine lung surfactant extract has been studied in order to try to shed light on the physicochemical bases underlying the potential adverse effects associated with pollutant inhalation. The surface pressure–area isotherms of lung surfactant (LS) films including increasing amounts of particles revealed that particle incorporation into LS monolayers modifies the organization of the molecules at the water/vapor interface, which alters the mechanical resistance of the interfacial films, hindering the ability of LS layers for reducing the surface tension, and reestablishing the interface upon compression. This influences the normal physiological function of LS as is inferred from the analysis of the response of the Langmuir films upon the incorporation of particles against harmonic changes of the interfacial area (successive compression–expansion cycles). These experiments evidenced that particles alter the relaxation mechanisms of LS films, which may be correlated to a modification of the transport of material within the interface and between the interface and the adjacent fluid during the respiratory cycle.

## Abstract 2

The inhalation of particulate matter in an urban environment is one of the most important sources of respiratory diseases. This makes it necessary to explore the potential impact of particles on the respiratory function for evaluating the potential damages associated with pollutant inhalation.

## Introduction

Long-term exposure to air pollutants is accounted among the most important sources of cardiovascular diseases and mortality.^1,2^ In particular, World Health Organization statistics ascribe more than one-third of the deaths caused by strokes, lung cancer, or cardiac diseases to air pollution.^3^ Therefore, air pollution must be considered a very important public health issue in industrialized society because of the continuous growth of pollutant emissions in the environment from different industries and combustion engines (power plants, vehicles, or heating systems).^4,5^

Among pollutants, fine particles are of particular importance in relation to their potential ability to induce acute health diseases.^6^ Fine particles can be inhaled and transported along the respiratory tract to the alveoli.^7,8^ Once particles arrive to the alveoli, they can interact with the lung surfactant (LS) complex, modifying their chemical composition and lateral organization. Furthermore, the interaction of LS and particles can favor the translocation of the latter toward other tissues and organs, including the bloodstream and the brain,^9,10^ which can induce an important series of adverse health effects.^11,12^ However, the current understanding of the potential biophysical changes associated with particle inhalation is far from clear. On one hand, the interaction of particles with LS alters their surface tension, phase behavior, and interfacial structure,^13,14^ whereas on the other hand particles are widespread in therapy and diagnostic.^15,16^

According to the above discussion, it is clear that LS emerges as one of the first biological barrier against inhaled pollutants and respiratory pathogens (e.g., SARS-CoV-2).^14,17−20^ LS is a complex mixture of lipids and proteins (mass ratio 9:1), which is placed as a thin liquid film overlying the inner alveolar walls, i.e., in the alveolar lining,^21−23^ and has as main functions the regulation of the surface tension of the alveoli, increase of lung compliance, and stabilization of the alveolar volume.^24^ These aspects present a critical impact on the normal physiological function of the lungs during respiration, and their modification can lead to different pathological situations, e.g., acute respiratory distress syndrome (ARDS) induced by the inhalation of particles.^25,26^ This makes necessary the design of *in vitro* models which can be used as preliminary assays for obtaining information on the modifications of the physicochemical properties of LS upon the incorporation of pollutants, limiting the use of invasive *in vivo* animal models.

The use of using interfacial sensitive techniques has emerged as a very promising approach for an *in vitro* an evaluation of the effect of different types of pollutants or particles on LS films.^27−32^ This type of study exploits many aspects related to the physical and chemical function of the LS depending on the unique properties of the liquid/vapor interface formed between the thin fluid film in the alveolar lining and the gas contained in the alveolar cavity.^33,34^

Many of the studies trying to elucidate how air pollutants affect the activity of LS are focused on the analysis of the impact of colloidal particles on monolayers containing only 1,2-dipalmitoyl-*sn*-glycero-3-phosphocholine (DPPC), which is the main component of the LS, accounting for about 50% in weight of the LS. DPPC plays a very important role in the reduction of the surface tension of the alveolar surface down to a quasi-null value during exhalation.^35−39^ However, the understanding of the function of the LS and the impact of the particles in its performance makes it necessary to use models containing, at least, most of the hydrophobic components of natural LS.^40^

The purpose of this study is to explore the interaction between hydrophobic silicon dioxide particles and a commercial formulation of porcine LS (Curosurf, Chiesi Farmaceutici S.p.A., Parma, Italy) using *in vitro* experimental assays based on the evaluation of the modification of the equilibrium and dynamic properties of LS films as a result of the incorporation of particles. The understanding of the impact of particles on the equilibrium properties of LS layers provides information on their ability for penetrating into LS films, and how they impact the packing of the molecules within the interface between the pulmonary fluid and the gas contained in the alveolar cavity. On the other hand, the evaluation of the effect of the particles on the dynamic response of LS films is important because most of the physiologically relevant aspects for the performance of LS are associated with its response upon periodical mechanical stresses. Previous studies based on the study of the interaction of hydrophobic silicon dioxide particles with minimal LS models based on DPPC monolayers have shown that the incorporation of particles modifies the packing of the molecules at the interface and their lateral cohesion, worsening the mechanical performance of the model LS films. This may induce a modification of the normal physiological function of the LS.^38,41,42^ This study tries to deepen the above-mentioned aspects by studying the effect of hydrophobic silicon dioxide particles on a more realistic LS model for understanding the potential impact of pollutants on the respiratory function. It is true that silicon dioxide particles are not commonly included among the most important urban pollutants. However, the role of the inhalation of silicon dioxide nanopowders in the emergence of silicosis makes it necessary to evaluate its effect on the performance of LS layers.^43^ Despite the simplicity of the experimental model used in this study, it may be expected that the obtained results can contribute to the understanding of the most fundamental physicochemical bases underlying the modification of the LS performance upon the incorporation of particles. This is of paramount importance because the impact of particles on the dynamic properties of fluid layers is a well-known problem from the seminal work of Lucassen^44^ that is more than 30 years old and has been studied by different authors beause of their multiple fundamental and applied implications.^45−47^

## Experimental Section

### Chemicals

Natural porcine extract (Curosurf) was a gift from Chiesi Farmaceutici S.p.A. (Parma, Italy). Curosurf was supplied as an aqueous dispersion (see composition in Table 1), which was lyophilized for obtaining a powder material before its use.^48,49^ Hydrophobic fumed silicon dioxide particles Aerosil R972 (SiO_2_) were purchased from Evonik-Degussa (Essen, Germany). These particles appear as chain-likes aggregates of primary particles with an average diameter of 16 ± 4 nm, a BET surface area of 110 m^2^/g, and a density of 2.2 g/cm^3^.^41^

**Table 1:** Composition of Commercial Curosurf (Sodium Chloride Solution)^50^

component	concentration (mg/mL)
phospholids, including:	76
-phosphatidylcholine	55 (30 mg/mL DPPC)
-acidid phospholids	6.40
-other	14.60
surfactant protein B	0.45
surfactant protein C	0.49
free fatty acids	0.55
triglycerides	0.10
cholesterol	0.02

Chloroform (CHROMASOLV, for high performance liquid chromatography, stabilized with ethanol) purchased from Sigma-Aldrich (Saint Louis, MO, USA) was used for preparing the spreading solutions of Curosurf and the dispersions of particles.

Ultrapure deionized water for cleaning and experiments was obtained with a multicartridge purification system Elix+Milli-Q (Millipore, Burlington, MA, USA). This water presents a resistivity higher than 18 MΩ cm, a total organic content lower than 6 ppm, and a surface around 72 mN/m at 22 °C without evidencing any surface tension kinetics water over several hours. The pH of the water was around 6.5, and no salts were used for fixing the ionic strength.

### Preparation of Monolayers

Curosurf monolayers at the water/vapor interface were obtained by dropping controlled volumes of Curosurf from a solution in chloroform (concentration about 1 mg/mL) using a high-precision Hamilton syringe (Hamilton Company, Reno, NV, USA). This methodology ensures the control of the interfacial density of the LS extract, Γ, upon solvent evaporation. The initial interfacial density of Curosurf spread at the water/vapor interface Γ_0_ was fixed in all of the experiments at a value of 0.16 μg/cm^2^.

Mixed monolayers containing the LS extract and the hydrophobic silicon dioxide particles were obtained following a two-step approach. First, a Curosurf monolayer is prepared by spreading the LS extract from its solution in chloroform (concentration 1 g/L) at the bare water/vapor interface, and then particles are spread from a dispersion in chloroform (concentration 1 g/L) onto the preformed LS monolayer to obtain mixed films with a defined particle mass fraction, again using chloroform as the spreading solvent. (Notice that particle dispersions were sonicated for 15 min using a laboratory ultrasound bath; this allows reducing the possible aggregation of the particles before their spreading). Once the monolayers (LS or particles + LS) are obtained, the interface is left for equilibration for 1 h before starting the experiments.

The temperature was fixed at 22.0 ± 0.1 °C in all of the experiments. Even though this temperature is far from the physiological one (37 °C), the main conclusions obtained in our study may be extrapolated, at least from a semiquantitative perspective, to the physiological conditions.

Further methodological information about the monolayer preparation can be found in the Supporting Information.

### Methods

A Langmuir trough KSV Nima model KN2002 (Biolin Scientific, Espoo, Finland), equipped with two Delrin barriers allowing for symmetric compression/expansion of the free liquid surface, was used for studying LS films under equilibrium and dynamic conditions. The surface tension, γ, was measured using a force balance fitted with a paper Wilhelmy plate (Whatman CHR1 chromatography paper, effective perimeter 20.6 mm, supplied by Sigma-Aldrich, St. Louis, MO, USA). The surface pressure, Π, is obtained as the difference between the surface tension of the pure water/vapor interface γ_w_ and γ, i.e., Π = γ_w_ – γ.

The quasi-equilibrium isotherms of pristine LS monolayers and upon the incorporation of particles were evaluated by measuring the change of the surface pressure, as the interfacial area available for the monolayer, *A*, is reduced at a fixed compression velocity of 2 cm^2^/min. This compression rate was found to be small enough for ensuring the absence of undesired nonequilibrium effects during the determination of the isotherms.^51^

The use of the Langmuir trough also allows for obtaining information related to the modifications of the response of LS monolayers to harmonic compression–expansion deformations of the interfacial area associated with the incorporation of particles, i.e., the response against dilational perturbations. This is possible by using the oscillatory barrier method, which is described elsewhere.^52,53^

The evaluation of the effect of particles in the response of the LS monolayers under conditions mimicking the respiratory cycle is possible by studying the dilational response of the monolayer upon nonlinear deformations at a surface pressure in the range 35–40 mN/m (highly condensed films).^54,55^ For this purpose, the rheological response of pristine LS films and LS layers upon the incorporation of silicon dioxide particles was evaluated for monolayers at a surface pressure in the range 35–45 mN/m upon deformations with the deformation amplitude *u* in the range 0.01–0.4 at a fixed frequency of 0.05 Hz.

Further experimental details can be found in the Supporting Information.

## Results and Discussion

In a previous work, it was demonstrated that the incorporation of hydrophobic silicon dioxide particles into a minimal LS model formed only by DPPC leads to a strong modification of the interfacial behavior due to the strong interaction of the particles with the hydrophobic moieties of the lipid molecules.^42^ This originates a strong disruption of the lipid packing at the interface, which in turn modifies the cohesion of the film and hence its ability for reducing the surface tension. In addition, this disruption of the film organization also leads to the modification of the relaxation mechanisms of the LS model upon compression–expansion deformations. Therefore, it may be expected that the incorporation of silicon dioxide particles can present a series of adverse consequences on the physiological function of LS, which can drive the emergence of different pathological situations. For a deeper understanding of the potential changes on the behavior of LS performance upon the incorporation of hydrophobic silicon dioxide particles, this work will follow the concepts of our previous study but replace DPPC for a porcine LS extract, containing all of the components involved in the performance of interfacial films of LS. This requires an analysis of the effect of different doses of particles on LS film performance. In particular, in this study the amount of particles incorporated into LS layers corresponds to estimated doses in the range 160–1440 μg/m^2^. These doses are between 80 and 800 times higher than the expected value for deposited particles on the alveolar surface after inhalation (around 1.9 μg/m^2^), and hence the results discussed in this work cannot be easily extrapolated to the true situation occurring under normal exposure to a polluted environment. However, this work can contribute to understanding the mechanisms responsible for the inactivation of LS after prolonged exposures to particles or after acute exposure events.^56−59^

### Understanding the Incorporation of Particles into LS Films: the Surface Pressure–Area Isotherm

**Figure 1:**
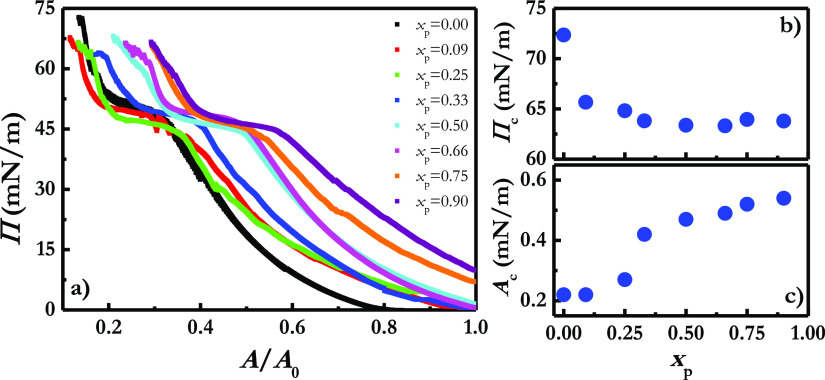
(a) Π–*A/A*_0_ isotherms for Curosurf monolayers upon the incorporation of different silicon dioxide particle mass fractions, *x*_p_. For the sake of comparison the isotherm corresponding to a pristine Curosurf monolayer is also reported. (b) Dependence of the collapse pressure, Π_c_, on the silicon dioxide particle mass fraction, *x*_p_, incorporated into the Curosurf monolayer. (c) Dependence of the area normalized in the maximum packing, *A*_c_, on the silicon dioxide particle mass fraction, *x*_p_, incorporated into the Curosurf monolayer.

The incorporation of particles into LS films as well as their impact on the organization of the molecules at the water/vapor interface can be evaluated in terms of the changes of the surface pressure–area isotherm (Π–*A* isotherm). It is true that from a biophysical perspective, the Π–*A* isotherms provide very limited information. However, these results provide important insights on how particles modify the cohesion of the molecules at the interface, and hence their lateral packing, which is important for deepening the origin of the potential adverse effects associated with particle inhalation.^60,61^Figure 1a displays the Π–*A* isotherms for LS films upon the incorporation of different particle mass fractions of silicon dioxide particles, *x*_p_. For the sake of comparison, the results corresponding to pristine LS monolayers is also represented in Figure 1a (notice that the area in Figure 1a appears normalized for a reference value *A*_0_ corresponding to the maximum area accessible for the interface before starting the experiment; therefore, the isotherms are shown as Π–*A/A*_0_ curves).

The compression isotherm obtained for the pristine Curosurf monolayer at the water/vapor interface is in good agreement with those previously reported for this commercial lung surfactant extract.^62,63^ Pristine Curosurf monolayers undergo a progressive increase of the surface pressure (i.e., decrease of the surface tension) with the compression (i.e., surface area reduction) up to a surface pressure of about 45 mN/m, where emerges a region characterized by an almost constant surface pressure value, i.e., a surface pressure plateau. This plateau corresponds to the monolayer-to-multilayer transition of the LS films and represents their equilibrium spreading surface pressure.^64^ Further reduction of the area available for the LS film leads to a rapid increase of the surface pressure, driving the LS film to the collapse for a surface pressure close to 72 mN/m (collapse surface pressure, Π_c_). This makes clear the ability of the LS layers for reducing the water/vapor surface tension upon compression to very low values (close to zero), which plays a very important role in normal respiratory function.

The incorporation of silicon dioxide particles into LS films does not modify the shape of the isotherm. However, particles lead to two important modifications on the interfacial phase behavior of Curosurf layers, with these modifications appearing more important as the mass fraction of silicon dioxide particles incorporated into the LS film is increased. The first modification induced by particle incorporation is the shifting of the Π–*A/A*_0_ isotherm to more expanded states; i.e., the incorporation of particles into the LS films leads to a prior lift-off of the surface pressure upon compression. It should be stressed that the film expansion cannot be ascribed to the surface activity of bare silicon dioxide particles which is negligible as was reported in our previous studies.^41,42^ Thus, the spread of the here used hydrophobic silicon dioxide particles in pristine water/vapor does not result in any significant change in the surface pressure, and the interfacial density of particles is high enough to ensure the formation of a layer with particles in close contact.

The second change associated with the presence of particles into the LS film is the reduction of the Π_c_ value (it can be understood better by analyzing the results in Figure 1b), which is related to a worsening of the ability of the LS for reducing the surface tension upon compression. It should be noted that even though the Π–*A/A*_0_ isotherms are determined under quasi-static conditions, which are far from what are expected for the respiratory cycle, the reduction of Π_c_ with the incorporation of particles emerges as preliminary evidence of the potential adverse impact of the particles in normal respiratory function.

A more detailed understanding of the impact of the incorporation of particles on the interfacial properties of LS layers requires a careful examination of the mechanistic bases underlying the modifications in the interfacial composition and the packing of the molecules. The incorporation of particles into the LS films drives the formation of a composite layer formed for the silicon dioxide particles and molecules of the LS. Thus, at a fixed value of the interfacial density of LS molecules, the incorporation of increasing silicon dioxide particle mass fractions into the LS monolayer leads to a situation in which the true interfacial density of the monolayer is higher than that corresponding to a pristine LS monolayer, and hence a part of the interfacial area available is occupied by particles. This induces an excluded area-like effect on the lateral packing of the LS at the water/vapor interface; i.e., the particles behave as obstacles to the monolayer packing. Therefore, the incorporation of particles leads to the LS film being in a situation equivalent to that corresponding to films at a higher compression degree (i.e., a lower value of *A*/*A*_0_) due to reduction of the area available for the organization of LS molecules.

The consideration of the excluded area-like effects alone provides a description of the shifting of the isotherm. However, it cannot account for the reduction of the collapse pressure with the incorporation of particles (see Figure 1b for clarity), which is associated with the lateral packing of the molecules at the water/vapor interface. Therefore, it should be expected that the incorporation of particles into the LS film drives a reduction of the cohesion between the molecules at the interface due to the emergence of hydrophobic interactions between the hydrophobic segments of lipids and proteins and the hydrophobic silicon dioxide particles. These interactions favor the retention of the particles at the LS film, but simultaneously they become obstacles for the reorientation of the LS molecules at the interface, which reduce the ability of LS films to form close packed layers, with high cohesion between the LS molecules. This limits the ability of the LS film for attaining quasi-null surface tension values, i.e., the reduction of the surface tension (increase the surface pressure), upon compression, and hence a premature alveolar collapse may be expected. This may be explained considering the limited effectiveness of the squeezing-out process of the silicon dioxide particles; i.e., particles cannot be not expelled from the interface, during the compression. The reduced effectiveness of the squeezing-out process emerges clear from the dependence of the area occupied by LS under maximum packing conditions, *A*_c_ (obtained by extrapolation of the linear part appearing at high surface pressure values of the Π–*A*/*A*_0_ curve to zero surface pressure), on the particle mass fraction incorporated into the monolayer, *x*_p_. Thus, the increase of *A*_c_ gives an indication of the quasi-irreversible trapping of the particles at the LS film due to the stabilizing role of the hydrophobic interactions between particles and the LS components (see Figure 1c). Therefore, it may be considered that the hydrophobic interactions are responsible for the stabilization and retention of hydrophobic particles within the LS film,^9^ which is very different from what happens for hydrophilic particles. The latter can be expelled from the LS monolayer upon compression, enabling the reduction of the surface tension down to values close to zero.^65^ On the other hand, the results show that the incorporation of hydrophobic particles into LS film beyond a critical mass fraction of about 0.3 does not lead to any significant modification either in the collapse pressure of the monolayer nor in *A*_c_, which may be associated with an aggregation of the silicon dioxide particles induced by the reduction of the area available. This leads to a reduction of the effective area occupied by each single particle, and consequently the particle effects remain similar to what is expected for monolayers with a lower mass fraction of incorporated particles. It should be noted that the above findings are compatible with those previously reported for the effect of the incorporation of the same particles studied in this work into simpler models formed by DPPC monolayers.^41,42^ This suggests that DPPC monolayers can provide important preliminary information about the impact of particles in the behavior of LS layers.

### Rigidification Induced by the Incorporation of Particles

**Figure 2:**
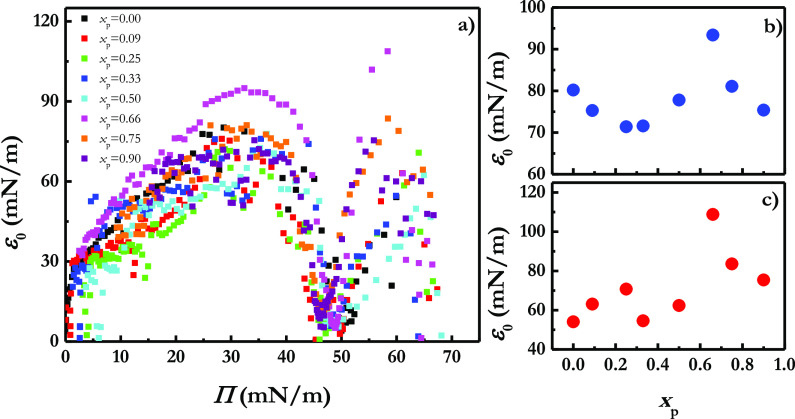
(a) ε_0_–Π relationships for Curosurf monolayers upon the incorporation of different silicon dioxide particle mass fractions, *x*_p_. For the sake of comparison, the curve corresponding to a pristine Curosurf monolayer is also reported. (b) Dependence of the maximum value of the quasi-equilibrium dilational elasticity on *x*_p_ before the plateau region. (c) Dependence of the maximum value of the quasi-equilibrium dilational elasticity on *x*_p_ after the plateau region.

The quasi-equilibrium dilational elasticity, ε_0_, can provide additional information about the effect of the incorporation of silicon dioxide particles on the cohesion of LS films in terms of the elastic energy stored by the monolayer during a continuous reduction of the interfacial area available, i.e., the rigidity of the monolayer, and can be obtained from derivation of the Π–*A* isotherm as^66^
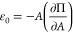
1Figure 2 displays the ε_0_–Π relationships obtained for LS film upon the incorporation of different silicon dioxide particle mass fractions. Independently of the particle mass fraction at the water/vapor interface, the dependences of ε_0_ on Π for Curosurf present similar shapes in agreement with the absence of any significant change on the Π–*A* isotherm. In general, LS films undergo an increase of their quasi-equilibrium dilational elasticity with compression to reach a maximum value, and then this drops down to a quasi-null value in the region corresponding to the surface pressure plateau of the isotherm. Once the plateau is overcome, ε_0_ starts to increase again with the compression up to the collapse point, which is followed for the film rupture and a new decrease of the elasticity.

For better understanding the impact of the particles in the quasi-equilibrium dilational elasticity, in Figure 2b and c are depicted the values corresponding to the maximum values of the quasi-equilibrium dilational elasticity before and after the coexistence region. Contrary to that what was previously reported for the interaction of hydrophobic particles with DPPC monolayers,^39,41,42,67^ the incorporation of increasing hydrophobic silicon dioxide particle mass fractions into LS monolayers leads to a rigidification of the interfacial film, i.e., the increase of ε_0_, at the lowest value of mass fraction of particles. This may be associated with a decrease of both the lateral mobility of the LS molecules within the interface and the flexibility of the lipid molecules,^9^ which may lead to a hindered packing of the LS film and, as a consequence, to reduction of the lateral cohesion. It is true that on the basis of the increase of ε_0_ with the incorporation of particles, the reduction of the cohesion appears counterintuitive. However, it may be understood considering that particles are obstacles avoiding the direct interaction between LS molecules. Therefore, the formation of plum-cake-like interfacial films may be expected where particles hamper the reorientation of the lipid molecules, which in turn worsens the fluidity of the LS film. The formation of a plum-cake like interfacial layer involves hydrophobic particles penetrating the LS layers toward the hydrophobic region, leading to the formation of LS films with insertions of silicon dioxide particles randomly distributed within their entire structure. This type of lateral organization of LS films upon particle incorporation agrees with the results obtained by Sosnowski et al.^68^ using molecular dynamics calculations.

The increase of the particle mass fraction beyond a threshold value around 0.66 leads to a decrease of the quasi-equilibrium dilational elasticity, which may be interpreted considering that even though the particles continue reducing the fluidity of the LS film, the increase of the number of particles incorporated within the interface can induce aggregation phenomena, which reduces the steric hindrance to the molecular mobility, and hence the fluidity of the LS films is partially restored. It should be noted that the increase of the quasi-equilibrium dilational elasticity found with the incorporation of particles is expected to be deleterious for lung recoil during the respiratory cycle.^34^

### Dilational Response of LS Films upon the Incorporation of Hydrophobic Silicon Dioxide Particles

The above discussion has evidenced that the incorporation of hydrophobic particles into LS films leads to an important modification of the organization of the molecules at the interface, which may alter their reorganization within the interface. Therefore, it is expected that the incorporation of particles into LS films can substantially modify the interfacial dynamics of LS, and, in particular, their relaxation dynamics. A first evaluation of the impact of particle incorporation in the relaxation of LS films can be obtained from the analysis of the response of the monolayer to a series of harmonic changes of the interfacial area (compression–expansion cycles) at fixed deformation amplitudes and frequencies. For the sake of example, the Supporting Information includes a pair of curves (see Figure S1a and b in Supporting Information) showing the traces of the deformation and response profiles obtained from oscillatory barrier experiments performed on an LS film with hydrophobic silicon dioxide particles at a fixed deformation amplitude within the linear response regime (*u* = 0.02).

The results show that the application of a sinusoidal deformation with small amplitude (within the linear regime) to LS monolayers leads to a sinusoidal surface pressure response with the same frequency. Furthermore, at this low amplitude, the strain–stress curve represented as a Lissajous plot (see Figure S1c in the Supporting Information) is found to be symmetric, which confirms the linear character of the response. Furthermore, the overlapping of the different compression–expansion cycles in the Lissajous plot evidence the good stability of the surface pressure response for deformations within the linear region. On the other hand, the Lissajous plot evidences a compression–expansion hysteresis, which may be explained considering the need of reorganization of the interfacial material upon expansion for reaching the same initial state (notice that results for other LS monolayers with different particle mass fractions are qualitatively analogous to those reported in Figure S1). Under the above conditions, it is possible to extract from the oscillatory barrier experiments information on the dependence of the viscoelastic dilational modulus (|*E*|) on the frequency (ν) (dilational relaxation spectrum). Figure S2 (see Supporting Information) displays some of the frequency dependences of the viscoelastic dilational modulus obtained by oscillatory barrier experiments on LS films with different hydrophobic silicon dioxide particle mass fractions at different values of reference surface pressure (the general features of the results obtained for other conditions are similar to those reported in Figure S2).

The dilational relaxation spectra (dilational viscoelastic modulus–deformation frequency curve) present an inflection point which can be ascribed to the characteristic frequency of a relaxation process occurring within the interfacial layer. The incorporation of hydrophobic silicon dioxide particles into the LS film leads to modification in the relaxation mechanism of the molecules at the interface, as is evidenced from the changes of the characteristic frequency evidenced on the experimental curves. For obtaining information on the characteristic relaxation frequency, the experimental data (see Figure S2 in the Supporting Information) can be analyzed using a rheological model assuming the existence of an interfacial relaxation process in an insoluble film according to the theoretical model provided by Ravera et al.^69^ Thus, it is possible to derive an expression accounting for the frequency dependence of the viscoelastic dilational modulus, which reads as follows
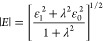
2where λ = ν_R_/ν, with ν_R_ being the characteristic relaxation frequency, and ε_0_ and ε_1_ are the low and high frequency limits of the dilational viscoelastic modulus within the explored frequency range, respectively. It should be noted that for insoluble monolayers, the ε_0_ values coincide with the quasi-equilibrium dilational elasticity obtained from the derivate of the isotherm (see Figure 2). Thus, the fitting of the experimental dependences of the viscoelastic dilational modulus on the deformation frequency to eq 2 using a nonlinear Levenberg–Marquardt least-squares fitting procedure by fixing ε_0_ to the value of the quasi-equilibrium dilational elasticity allows obtaining of the values of the characteristic relaxation frequency of the relaxation processes, ν_R_, and ε_1_. Selected theoretical curves obtained using the model defined by eq 2 are displayed in Figure S2 together with the experimental results.

**Figure 3:**
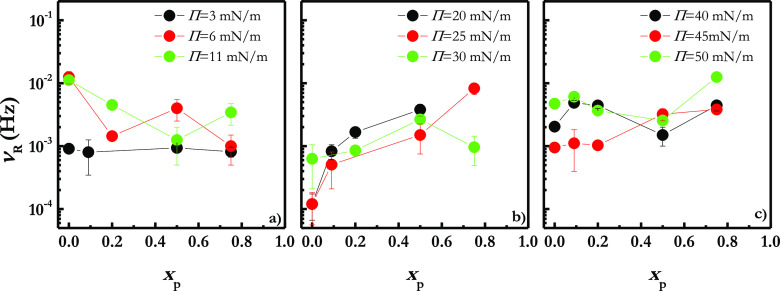
(a) Characteristic relaxation frequencies, ν_R_, for LS films with different hydrophobic silicon dioxide particle mass fractions, *x*_p_, as were obtained using the oscillatory barrier method at three different values of reference surface pressure: 3, 6, and 11 mN/m. (b) Characteristic relaxation frequencies, ν_R_, for LS films with different a hydrophobic silicon dioxide particle mass fractions, *x*_p_, as were obtained using the oscillatory barrier method at three different values of reference surface pressure: 20, 25, and 30 mN/m. (c) Characteristic relaxation frequencies, ν_R_, for LS films with different hydrophobic silicon dioxide particle mass fractions, *x*_p_, as were obtained using the oscillatory barrier method at three different values of reference surface pressure: 40, 45, and 50 mN/m.

Figure 3 reports the dependence of the characteristic relaxation frequency ν_R_ of the dilational response on the mass fraction of particles incorporated into the LS films for different reference values of the surface pressure. From the results, it is clear that the hydrophobic silicon dioxide particles induce direct changes in the activity of the LS films from the lowest concentrations. The modification of the interfacial dynamics of LS films associated with the incorporation of particles emerges dependent on the particle mass fraction and the reference surface pressure state, i.e., the interfacial density of the monolayer, which agrees with the results of Kondej and Sosnowski.^70^

In the region of low surface pressure (below 20 mN/m), it is found that the incorporation of increasing particle mass fractions leads to a reduction of the characteristic relaxation frequency (in the range 10^–3^ to 10^–2^ Hz), i.e. the motion of the molecules becomes slower. This may be an additional signature of the rigidification induced by the nanoparticles of the LS film, which makes the packing of the molecules at the interface more difficult. Furthermore, the characteristic relaxation frequency increases with the value of the surface pressure of the reference state, which may be associated with the aggregation of the particles as the layer becomes more compacted. This makes possible a better reorganization of the molecules within the monolayers.

For monolayers at surface pressures of 20 mN/m and above, the effect of the rigidification is less in evidence, and the characteristic relaxation frequency (in the range 10^–3^ to 10^–2^ Hz) increases as the particle mass fraction is increased. This unexpected behavior may only be understood by considering that the incorporation of hydrophobic silicon dioxide particles into LS films leads to the emergence of a very complex rheological response. Thus, the existence of a plum-cake-like distribution of LS molecules and particles makes possible the existence of coupled interfacial dynamics. Furthermore, the steric hindrance interactions induced by the particles may contribute to the emergence of complex rearrangements within the interface, which may involve both the particles and the LS molecules. In particular, it may be expected that the higher the interfacial density of particles (i.e., higher *x*_p_), the higher the particle aggregation, making possible a faster reorganization of the LS molecules within the interface.^70^ Therefore, the incorporation of particles into LS films modifies the relaxation mechanism by combination of two counteracting effects: (i) rigidification induced by the particle incorporation and (ii) aggregation of the particles at the LS film. The former slows down the molecular reorganization at the interface, emerging as dominant at the lowest values of surface pressure, whereas the aggregation of particles has the opposite effect, enhancing the motion of the molecules at the interface, and increases its importance as the surface pressure and particle mass fraction at the interface is increased. It should be noted that the relaxation times found for pristine LS films and upon the incorporation of particles are larger than that expected for molecular species. However, this surprising finding may be rationalized considering that the strong lateral interactions between lipids, proteins, and particles hinder the lateral motion of the molecules at the interface, which in turn leads to their relaxation at very long times in agreement with the theoretical model proposed by Arriaga et al.^71^

**Figure 4:**
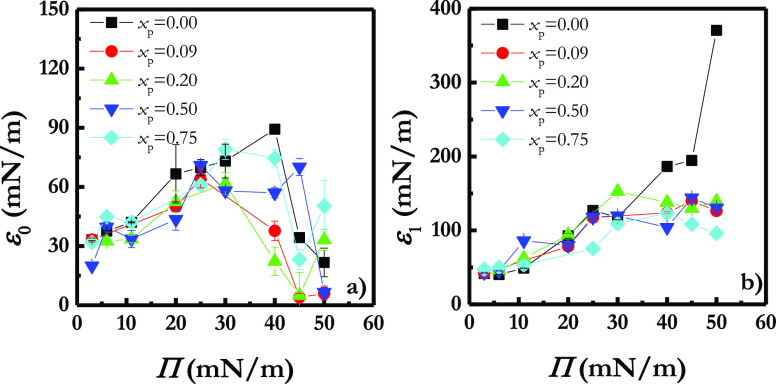
Surface pressure dependences of the low, ε_0_, and high, ε_1_, frequency limits of the dilational viscoelastic modulus for LS films upon the incorporation of different hydrophobic silicon dioxide particle mass fractions *x*_p_.

The incorporation of hydrophobic silicon particles into the LS films also modifies the low and high frequency limits of the dilational viscoelastic modulus. Figure 4 displays the dependence of the low and high frequency limits of the dilational viscoelastic modulus on the surface pressure obtained from the analysis of the experimental mechanical spectra using the model described for eq 2.

The dependence of the limit values of the dilational viscoelastic modulus agrees with the above discussion on the quasi-equilibrium elasticity. As expected, the data of ε_0_ depicted in Figure 4a show the same dependences discussed above for the quasi-equilibrium dilational elasticity. More interestingly, the dependence of ε_1_ on *x*_p_ displayed in Figure 4b emerges. The incorporation of particles into the LS film reduces the deformability of the monolayers; i.e., the value of ε_1_ is reduced, which is an additional confirmation of the rigidification induced for the particles.

### Mimicking the Respiratory Cycle

**Figure 5:**
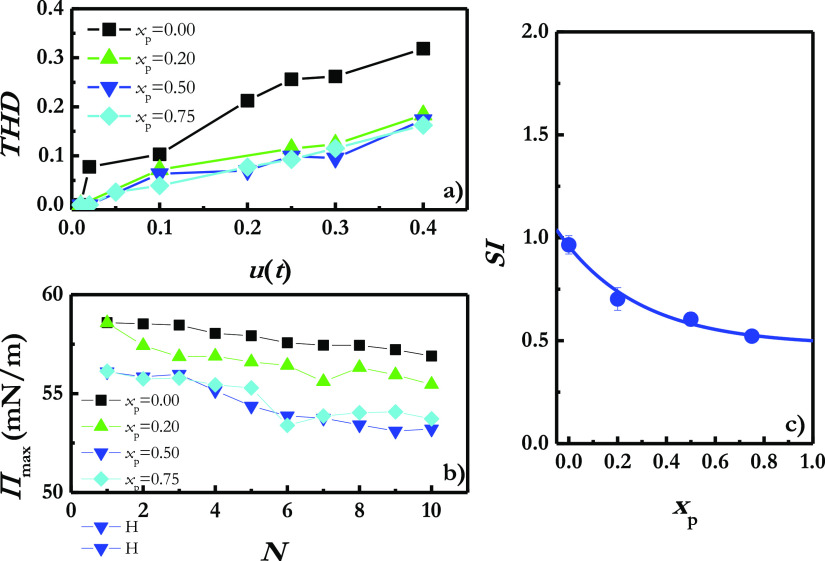
(a) Dependence of the total harmonic distortion calculated from the data in the FFT spectra using eq 3 on the deformation amplitude for a monolayer of LS with the incorporation of different particle mass fractions. (b) Dependence of the Π_max_ on the number of compression–expansion cycles for LS films upon the incorporation of different particle mass fractions. (c) Dependence of SI on the particle mass fraction incorporated into the LS film. The data correspond to the average over 10 compression–expansion cycles, and the error bars represent the standard deviation. Data in all panels correspond to experiments around a reference surface pressure in the range 35–45 mN/m and deformations with *u* = 0.40 and ν = 0.05 Hz, which are typical conditions that allow mimicking the respiratory cycle. In all of the panels, the lines are guides for the eyes.

A better understanding of the true biophysical impact of the incorporation of particles into LS films requires an analysis of the response of the monolayers upon harmonics changes of the interfacial area at deformations of about 40% of the total interfacial area around a surface pressure in the range 35–45 mN/m, which is assumed to be the surface pressure inside static alveoli.^54,55^ At first approach, the effect of the incorporation of particles into LS films was analyzed for successive compression–expansion cycles with amplitudes of deformation in the *u* range 0.01–0.40 and a fixed deformation frequency of 0.05 Hz. For obtaining information on such experiments, which are expected to drive the LS films to the region of nonlinear response as the deformation amplitude is increased (for the sake of example, Figure S3a and c in the Supporting Information show the Lissajous plots for monolayers upon deformation in the linear and nonlinear regimes, respectively), the stress response was initially analyzed by applying a Fast Fourier Transform algorithm (FFT).^72,73^ For deformations within the region of linear response, the FFT spectra lead only to a peak corresponding to the fundamental frequency, whereas the overtones of the fundamental frequency start to appear once the amplitude of deformation goes beyond the onset on the region of nonlinear response (for results see Figure S3b and d in the Supporting Information, where the FFT spectra for responses within the linear and nonlinear regimes, respectively, are displayed). Further details on this methodology for the analysis of the compression–expansion data may be found in refs (36) and (53). The evaluation of the nonlinear character of the response can be accounted by introducing the total harmonic distortion (THD) defined as^74^
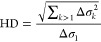
3with Δσ_1_ being the amplitude corresponding to the fundamental oscillation frequency and Δσ_*k*_, the frequency of the different overtones of the fundamental oscillation frequency (*k* ≠ 1). For systems with a linear rheological response, only the peak corresponding to the fundamental oscillation frequency is expected in the FFT spectrum (see Figure S3b in the Supporting Information), and hence the THD assumes a null value, whereas values larger than unity are found for systems in which the response becomes nonlinear appearing peaks with *k* > 1 in the FFT spectrum. Therefore, the evaluation of the THD value provides insightful information for characterizing the linearity or nonlinearity of the rheological response at different deformation amplitudes of LS films upon the incorporation of silicon dioxide particles. Figure 5a displays the dependence of the THD on deformation amplitude (*u*) for LS films and for LS films upon the incorporation of different hydrophobic silicon dioxide particle mass fractions.

The increase of the amplitude of the deformation of the interfacial area pushes the response of the monolayer toward the nonlinear region. However, more interesting is the analysis of the impact of the particles on the linearity of the response of LS films. The results show that the incorporation of particles into LS films enhances the linear character of the rheological response, i.e., reduces the THD value. This is the opposite situation of that which was found for heterogeneous systems such as LS-particle composite films^71^ and deserves a careful analysis.

The weakening of the nonlinear character of the rheological response as a result of the particle incorporation may be considered a result of the modification of the transport mechanism occurring within the interface and between the interface and the fluid phase, which alters the composition clearance of LS films upon harmonic compression–expansion deformations of the area available. Therefore, considering that the incorporation of particles reduces the fluidity of the LS layers, a change may be expected on the mechanism driving the reorganization of the molecules and consequently the interfacial dynamics, which modifies the mechanical response under biophysically relevant conditions. This can lead to an important impact on the normal physiology of LS films. Further details on the effect of particles on the physiological performance of LS films can be obtained from the analysis of the maximum surface pressure Π_max_ (corresponding to the minimum surface tension) that is reached in the successive compression–expansion cycles performed in the monolayer under conditions close to those expected for a normal respiratory cycle (surface pressure in the range 35–45 mN/m, *u* = 0.40 and ν = 0.05 Hz). Figure 5b displays the dependence of Π_max_ on the number of cycles *N* for LS monolayers upon the incorporation of different hydrophobic silicon dioxide particle mass fractions.

The results evidence that the maximum value of Π_max_ is reached for pristine LS monolayers, whereas the incorporation of particles leads to a decrease of the value of Π_max_. This is in agreement with the above discussion about the inactivation of the LS functionality upon the incorporation of particles. Furthermore, the incorporation of particles leads to a decrease of the Π_max_ with the number of cycles, which may be associated with an impoverishment of the interfacial layers on surface active molecules. This probably occurs due to a partial adsorption of some lipid and proteins molecules onto the particles surface, which leads to a noneffective clearance process of the particles from the interface during compression and reduces the effectiveness of the respreading during the expansion of the interfacial area of the material expelled as a result of the compression. This is related to the progressive damage on the interfacial properties of the LS, which can be associated with an acute respiratory disease.

The inactivation of the interfacial properties of the LS layers as a result of the incorporation of particles is confirmed by analyzing the size of the stress response (Π_max_ – Π_min_, with Π_min_ being the minimum surface pressure reached during the expansion) for compression–expansion cycles within the physiologically relevant range as shown Figure S4 in the Supporting Information. The reduction of the size of the compression–expansion loop upon the incorporation of particles confirms the worsening of the surface activity of the LS film, which in turn may alter its functionality. This is also supported by the strong decrease of the stability index SI (see Figure 5c) defined as^75^
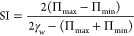
4Therefore, the results evidence that beyond the ability for surface tension reduction, the incorporation of particles leads to the emergence of different effects on the stability of the respiratory cycle, which can affect the normal respiratory function. In particular, the decrease of SI, which evaluates the change of the surface tension during a compression–expansion cycle, indicates that the incorporation of particles into LS film leads the systems toward a pathological state, characterized for a lower variation of the surface tension during the respiratory cycle, and thus resulting in a partial inactivation of LS.

In summary, the above results have evidenced that the incorporation of hydrophobic silicon dioxide particles into LS films worsens the ability of the LS for reducing the surface tension (increase surface pressure), which should be considered an important signature of the reduction of the alveolar stability. This together with the reduction of the fluidity of the LS film may lead to a premature alveolar collapse and respiratory failure. Therefore, modification of the organization of the molecules at the interface induced by the incorporation of the particles modifies the dynamic response of the LS films, in such a way that it emerges strongly dependent on the particle mass fraction and the aggregation state of the particles. The latter controls the impact of particles on the relaxation mechanism of the LS films, counteracting the reduction of the molecular mobility emerging from the rigidification of the LS film. The change of the relaxation mechanism of the layers upon the incorporation of particles leads to a situation in which the LS performance starts to be inactivated, and hence a dysfunctional behavior may be expected during the respiratory cycle associated with the modification of the physiological mechanisms of mass transport. The here presented results allow us to infer that inhaled hydrophobic silicon dioxide particles may induce important health problems as a result of the emergence of specific physicochemical phenomena in the lung surface, which can impact the normal respiratory dynamics. Therefore, the analysis of the impact of hydrophobic silicon dioxide particles on the interfacial properties on the LS film has provided very valuable information for a preliminary analysis of the potential risks associated with the inhalation of the particles. In particular, those risks are associated with the dysfunction/inactivation of LS after prolonged exposure to particles (i.e., particle accumulation in the lungs) or after acute exposure events (e.g., (occupational or accidental inhalation of ultrahigh doses).

## Supporting Information Available

Experimental details; examples of traces for deformation and stress response for oscillatory barriers experiments within the regions of linear and nonlinear response; examples of FFT spectra of stress response for oscillatory barriers experiments within the regions of linear and nonlinear response; representative curves of the dependences of the viscoelastic modulus on deformation frequency; surface pressure change for compression–expansion of the interfacial are under conditions mimicking the respiratory cycle (PDF)
