# Supplementary material for: Evaluating the Impact of Hydrophobic Silicon Dioxide in the Interfacial Properties of Lung Surfactant Films
Source: Environ Sci Technol. 2022 Jan 26;56(11):7308–18. doi: 10.1021/acs.est.1c06885 (PMC9178919; doi:10.1021/acs.est.1c06885)
Supplement: Supplementary file 1 — es1c06885_si_001.pdf [file es1c06885_si_001.pdf]

Supporting Information for

Evaluating the impact of hydrophobic silicon dioxide in the interfacial properties of lung surfactant films

Eduardo Guzmán,[1,2,*] Eva Santini,[3] Michele Ferrari,[3] Libero Liggieri,[3] Francesca Ravera[3,*]

[1] Departamento de Química Física, Facultad de Ciencias Químicas, Universidad Complutense de Madrid. Ciudad Universitaria s/n, 28040-Madrid (Spain)

[2] Instituto Pluridisciplinar, Universidad Complutense de Madrid. Paseo de Juan XXIII 1, 28040-Madrid

[3] Istituto di Chimica della Materia Condensata e di Tecnologia per l'Energia, UOS Genova-Consiglio Nazionale delle Ricerche (ICMATE-CNR). Via De Marini 6, 16149-Genova (Italy)

[*] Corresponding authors:
Eduardo Guzmán. Departamento de Química Física, Facultad de Ciencias Químicas, Universidad Complutense de Madrid. Ciudad Universitaria s/n, 28040-Madrid (Spain) Phone: +34 91 394 4107. e-mail: eduardogs@quim.ucm.es

Francesca Ravera. Istituto di Chimica della Materia Condensata e di Tecnologia per l'Energia, UOS Genova-Consiglio Nazionale delle Ricerche (ICMATE-CNR). Via De Marini 6, 16149-Genova (Italy). Phone: Phone +39 010 647 5725. e-mail: francesca.ravera@ge.icmate.cnr.it

TABLE OF CONTENTS:

	Page
S.1. Experimental details	S.2
S.1.1. Preparation of monolayers	S.2
S.1.2. Methods	S.3
S.2. Results	S.6
S.2.1. Dilational response of LS films upon the incorporation of hydrophobic silicon dioxide particles	S.6
Figure S.1. Deformation and stress response profiles for an oscillatory barrier experiment	S.6
Figure S.2. Elastic modulus versus frequency curves for selected experiments	S.7
S.2.2. Mimicking the respiratory cycle	S.8
Figure S.3. Experimental traces and their respective FFT for oscillatory barrier experiments within the regions of linear and non-linear response	S.8
Figure S.4. Amplitude of the surface pressure change under deformations mimicking the respiratory cycle	S.9

S.1. Experimental details

S.1.1. Preparation of monolayers. Curosurf® monolayers at the water/vapor interface were obtained by dropping controlled volumes of Curosurf® from a solution in chloroform (concentration about 1 mg/mL) using a high-precision Hamilton syringe (Hamilton Company, Reno, NV, USA). This methodology ensures the control of the interfacial density of the LS extract, Γ, upon solvent evaporation. The initial interfacial density of Curosurf® spread at the water/vapor interface Γ_0 was fixed in all the experiments in a value of 0.16 μg/cm^2.

Mixed monolayers containing the LS extract and the hydrophobic silicon dioxide particles were obtained following a two-step approach. First a Curosurf® monolayer is prepared by spreading the LS extract from its solution in chloroform (concentration 1 g/L) at the bare water/vapor interface, and then a given amount of the particle dispersion (concentration 1 g/L) is spread onto the preformed LS monolayer, again using chloroform as the spreading solvent (Notice that particles dispersions were sonicated during 15 min using a laboratory ultrasound bath; this allows reducing the possible aggregation of the particles before their spreading). The use of this methodology for the incorporation of particles into the LS film allows tuning almost at will the LS:particles weight ratio at the interface evaluated in terms of the weight fraction of particles at the interface x_{p}. Once the monolayers (LS or particles+LS) are obtained, the interface is left for equilibration during 1 h before starting the experiments. This time was found to be enough for ensuring a complete evaporation of the chloroform and, in the particular case of the mixed monolayer, for the achievement of the equilibrium of the composite system driven by the LS–particle interactions [1, 2]. It should be noted that the monolayers containing LS and silicon dioxide particles cannot be strictly defined as mixed monolayer because they were not obtained by direct co-spreading of a mixed dispersion containing both LS and silicon dioxide particles (first, the LS was spread at the pristine water/vapor interface, and then the addition of the particles is made onto the preformed LS monolayer). However, for the sake of simplicity, the term mixed monolayers will be used in this work for referring to monolayers involving LS and particles. It should be noted that the methodology used for the incorporation of particles into the LS films can modify the obtained results. However, the general conclusions extracted from the experimental data appears rather independently of the methodology followed for the mixed film preparation [3]. In this work, it has been followed a methodology in which the interaction between particles and

LS layers occurs only at the water/vapor interface, which may be consistent with that what happens during the *in vivo* interaction between environmental pollutants and LS in the alveoli. However, this methodology cannot include two important aspects that may have impact when *in vivo* conditions are considered: (i) the presence of chloroform during particles addition may alter both the lateral packing of the molecules at the interface and the LS–particle interactions [3], and (ii) the interaction of particles and LS may be affected for specific mass transport boundary conditions which cannot be included in studies done in Langmuir troughs [4, 5].

S.1.2. Methods. A Langmuir trough KSV Nima model KN2002 (Biolin Scientific, Espoo, Finland), equipped with two Delrin® barriers allowing for symmetric compression/expansion of the free liquid surface, was used for studying LS films under equilibrium and dynamic conditions. The total surface area of the teflon trough is 243 cm^2. The surface tension, γ, was measured using a force balance fitted with a paper Wilhelmy plate (Whatman CHR1 chromatography paper, effective perimeter 20.6 mm, supplied by Sigma Aldrich, St. Louis, MO, USA), ensuring a zero contact angle. The surface pressure, Π, is obtained as the difference between the surface tension of the pure water/vapor interface γ_w and γ, i.e. $\Pi = \gamma_w - \gamma$.

The quasi-equilibrium isotherms of pristine LS monolayers and upon the incorporation of particles were evaluated by measuring the change of the surface pressure as the interfacial area available for the monolayer, A, is reduced at a fixed compression velocity of 2 cm^2/min, which is equivalent to a compression rate $(\Delta A/A_0)/\Delta t$ of about 10^{-5} s^{-1}, with $\Delta A/A_0$ being the amplitude of the deformation, represented as the ratio between the change of area during the compression ΔA, and the reference interfacial area A_0 (generally the area in which the compression is started), and Δt the time needed for the deformation. This compression rate was found to be small enough for ensuring the absence of undesired non-equilibrium effects during the determination of the isotherms [6].

The use of the Langmuir trough also allows obtaining information related to the modifications of the response of LS monolayers to harmonic compression-expansion deformations of the interfacial area associated with the incorporation of particles, i.e., the response against dilational perturbations. This is possible by using the oscillatory barrier method which is described elsewhere [7, 8]. For this purpose, it was studied the relaxation mechanisms against periodic changes of the area available for monolayers of pristine LS

films and upon the incorporation of different particle weight fraction at different references states defined by different values of the surface pressure. This requires to evaluate the dilational viscoelastic modulus upon deformations of different frequencies (in the range 10^{-3}-0.15 Hz), and a fixed amplitude of deformation within the region of linear response of the monolayer.

The oscillatory barrier method relies on the evaluation of the complex dilational viscoelastic modulus $|E| = \Delta\gamma/(\Delta A/A)$, which is related to the variation of the surface tension γ as response to a harmonic change of the interfacial area at a controlled frequency ν.This harmonic change of the interfacial area can be define in terms of the following sinusoidal function

$$A(t) = A_0 + \Delta A sin(2\pi\nu t). \tag{S1}$$

The harmonic change of the interfacial area (strain) results in a stress response $\Delta\Pi = \Pi_0 - \Pi(t)$, which is defined as the different between the surface pressure of a reference state defined for Π_0 and their instantaneous value $\Pi(t)$. For deformations of small amplitude, i.e. deformation within the linear regime, it may be expected that the stress response can also follow a sinusoidal profile characterized for the same frequency than the strain

$$\Pi(t) = \Delta\Pi sin(2\pi\nu t + \phi). \tag{S2}$$

with ϕ accounting for the emergence of a possible delay on the stress response in relation to the strain, which is associated with the viscous contribution to the interfacial response. For systems having a linear response, the stress includes two terms, the elastic term proportional to the instantaneous deformation $u(t) = A(t) - A_0/A_0$ and the viscous one which depends on the deformation rate $du(t)/dt$, and can be written as

$$\Pi(t) = \varepsilon' u(t) + \eta(du(t)/dt), \tag{S3}$$

where ε' and η represents the dilational elasticity and viscosity, respectively. The complex dilational viscoelasticity can be obtained from Equation (S3) by assuming a generic harmonic perturbation

$$|E| = \varepsilon' + 2\pi\nu\eta\text{i}, \tag{S4}$$

with i=$(-1)^{1/2}$. The analysis of the curves corresponding to the strain and stress in terms of Equations (S1) and (S2) allows obtaining information related to the amplitude and

phase shift, which makes possible the calculation of the dilational viscoelastic modulus. It should be noted that for the here reported experiments, an amplitude of the dilational deformation u=0.02 was adopted. This provides information related to the response of the monolayers within the linear regime. It should be noted that this type of experiments provides very helpful information for analyzing the impact of incorporation of particles on the relaxation mechanisms leading to the equilibration of LS lipid layers, which has been demonstrated as a very useful tool for exploring the complex dynamic situations that are found in LS layers [9, 10]. However, the understanding of the effect of particles upon compression-expansion of the interfacial area mimicking that what occur during the respiratory cycle requires to push the system beyond the linear regime [11], i.e. the increase of the amplitude of the dilational deformation. This is also possible by using the oscillatory barrier method in a Langmuir trough. However, the studies focused on evaluating the response of pristine LS monolayers and upon the incorporation of particles are not aimed to obtain information of the viscoelastic dilational modulus.

The evaluation of the effect of particles in the response of the LS monolayers under conditions mimicking the respiratory cycle makes necessary to study the dilational response of the monolayer upon non-linear deformations (u in the range of 0.3–0.4) with a frequency in the range 0.04-0.2 Hz [11, 12] at a surface pressure in the range 35-40 mN/m (highly condensed films). For this purpose, the rheological response of pristine LS films and upon the incorporation of silicon dioxide particles was evaluated for monolayers at a surface pressure in the range 35-45 mN/m, which is commonly associated with the surface pressure in a static alveoli, upon deformations with u in the range 0.01-0.4 at a fixed frequency of 0.05 Hz. This type of studies provides information on the effect of the particle incorporation on the mechanical behavior of LS layers upon conditions close to that what are found during the respiratory cycle.

S.2. Results

S.2.1. Dilational response of LS films upon the incorporation of hydrophobic silicon dioxide particles.

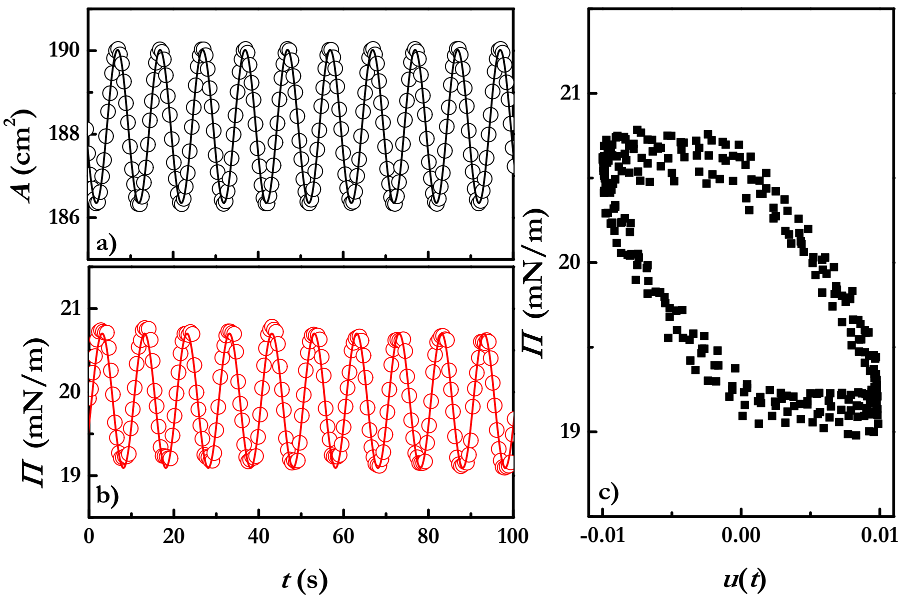

Figure S1. (a) Deformation profile for an oscillatory barrier experiment ($u(t)$=0.02 and ν=0.1 Hz) corresponding to a monolayer of LS with silicon dioxide particles (x_p=0.75) at a reference state of 20 mN/m. The line represents the calculated profile obtained using Equation (1). **(b)** Surface pressure response profile for the monolayer of panel **(a)**. The line represents the calculated profile obtained using Equation (1). **(c)** Lissajous plot corresponding to the deformation and surface pressure response profiles of panels **(a)** and **(b).** Notice that the Lissajous plot shows the results corresponding to 12 compression-expansion cycles.

For obtaining information on the characteristic relaxation frequency from the dependences of the viscoelastic modulus on the deformation frequency, the experimental data can be analyzed in terms of a model considering the existence of an interfacial relaxation process in an insoluble film, which defines the frequency dependence of the viscoelastic dilational modulus as [13]

$$|E| = \left[\frac{\varepsilon_1^2 + \lambda^2\varepsilon_0^2}{1+\lambda^2}\right]^{\frac{1}{2}}, \qquad \text{(S5)}$$

where $\lambda = \nu_R/\nu$, with ν_R being the characteristic relaxation frequency, and ε_0 and ε_1 are the low and high frequency limits of the dilational viscoelastic modulus within the explored frequency range, respectively.

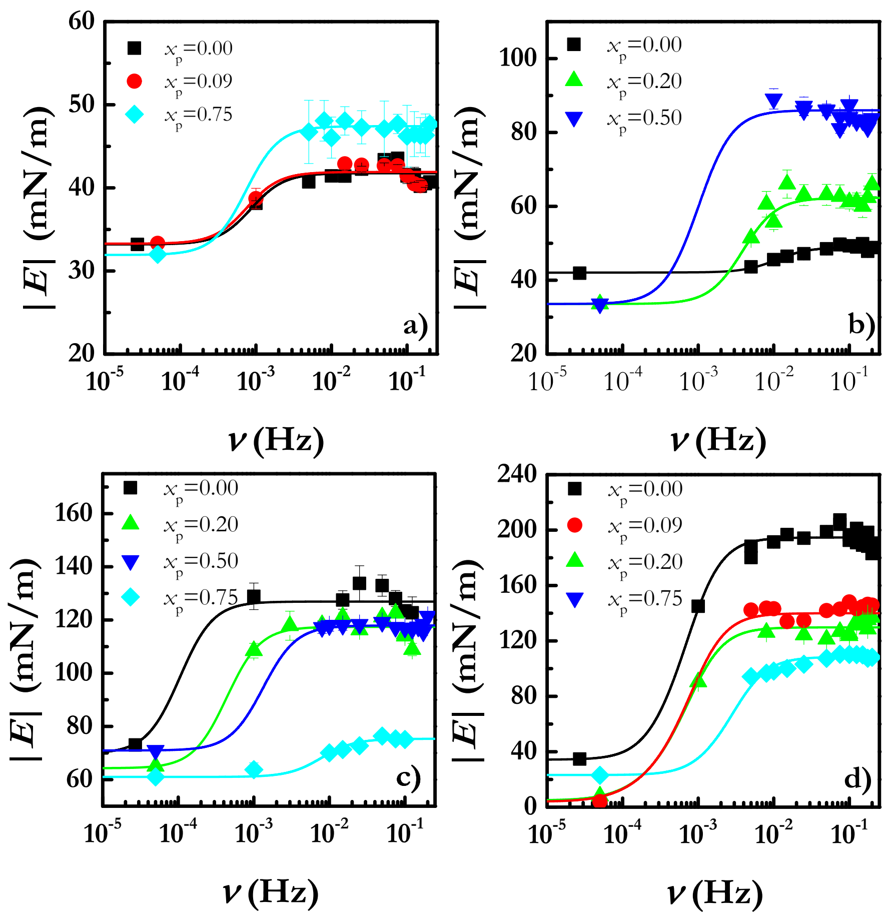

Figure S2. Dependence of the modulus of the dilational viscoelasticity, $|E|$ on the deformation frequency, ν, obtained by the oscillatory barrier method for LS films upon incorporation different hydrophobic silicon dioxide particle mass fraction, x_p, at different values of the reference surface pressure Π. **(a)** Π=3 mN/m. **(b)** Π=11 mN/m. **(c)** Π=25 mN/m. **(d)** Π=45 mN/m. The symbols and the lines represent the experimental data and the curves obtained from their modelling using Equation (S5), respectively.

S.2.2. Mimicking the respiratory cycle

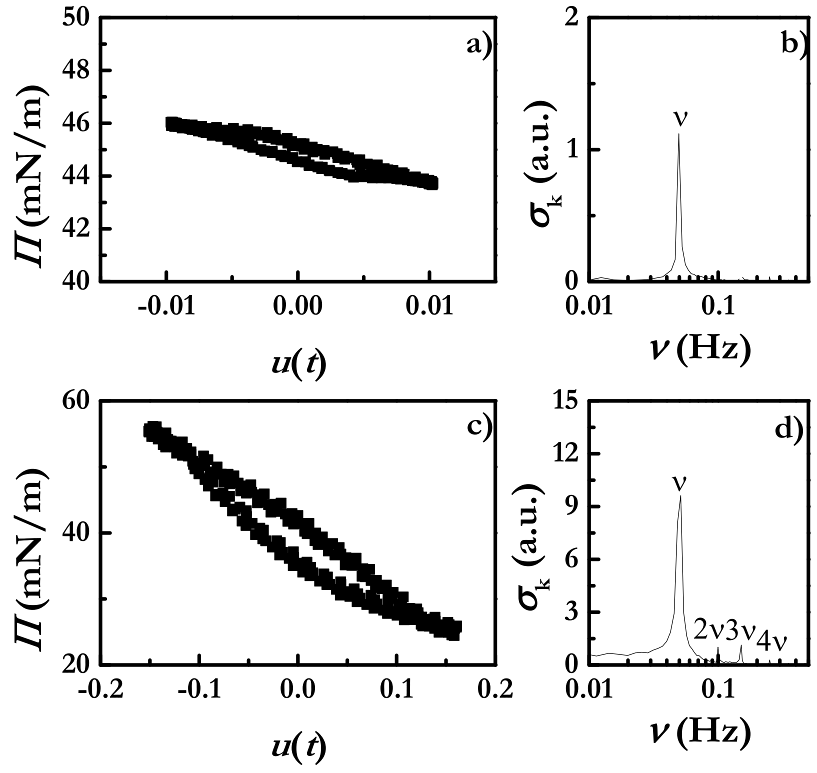

Figure S.3. (a) Lissajous plot corresponding to the deformation and surface pressure response profiles of LS film with a weight fraction of particles of 0.75, and a deformation within the linear regime ($u(t)$=0.02 and ν=0.05 Hz). Notice that the Lissajous plot shows the overlapping of 5 compression-expansion cycles. **(b)** FFT spectrum for the data in panel **(a)**. Notice that the FFT spectrum only shows the signal corresponding to fundamental frequency. **(c)** Lissajous plot corresponding to the deformation and surface pressure response profiles of LS film with a weight fraction of particles of 0.75, and a deformation within the non-linear regime ($u(t)$=0.32 and ν=0.05 Hz). Notice that the Lissajous plot shows the overlapping of 5 compression-expansion cycles. **(d)** FFT spectrum for the data in panel **(c)**. Notice that the FFT spectrum shows the signal corresponding to fundamental frequency and several overtones. Data in all panels correspond to experiments around a reference surface pressure in the range 35-45 mN/m, which is the typical surface pressure considered for static alveoli.

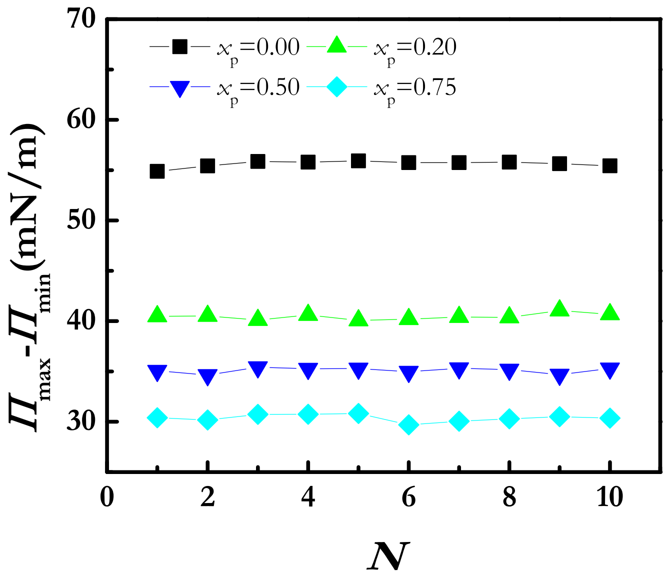

Figure S4. Dependence of Π_{max}-Π_{min} on the number of compression-expansion cycles for LS films upon the incorporation of different particle mass fraction. Data correspond to experiments around a reference surface pressure in the range 35-45 mN/m and deformations with $u(t)$=0.40 and ν=0.05 Hz, which are typical conditions that allows mimicking the respiratory cycle. The lines are guides for the eyes.

References

[1] Guzmán E, Santini E, Ferrari M, Liggieri L, Ravera F. Interfacial Properties of Mixed DPPC–Hydrophobic Fumed Silica Nanoparticle Layers. J Phys Chem C. 2015;119:21024-34.

[2] Guzmán E, Santini E, Zabiegaj D, Ferrari M, Liggieri L, Ravera F. Interaction of Carbon Black Particles and Dipalmitoylphosphatidylcholine at the Water/Air Interface: Thermodynamics and Rheology. J Phys Chem C. 2015;119:26937-47.

[3] Miguel Diez M, Buckley A, Tetley TD, Smith R. The method of depositing CeO2 nanoparticles onto a DPPC monolayer affects surface tension behaviour. NanoImpact. 2019;16:100186.

[4] Gradoń L, Podgórski A, Sosnowski TR. Experimental and Theoretical Investigations of Transport Properties of DPPC Monolayer. J Aerosol Med 1996;9:357-67.

[5] Gradoń L, Podgórski A. Hydrodynamical model of pulmonary clearance. Chem Eng Sci. 1989;44:741-9.

[6] Hifeda YF, Rayfield GW. Evidence for first-order phase transitions in lipid and fatty acid monolayers. Langmuir. 1992;8:197-200.

[7] Mendoza AJ, Guzmán E, Martínez-Pedrero F, Ritacco H, Rubio RG, Ortega F, Starov VM, Miller R. Particle laden fluid interfaces: Dynamics and interfacial rheology. Adv Colloid Interface Sci. 2014;206:303-19.

[8] Guzmán E, Liggieri L, Santini E, Ferrari M, Ravera F. Influence of silica nanoparticles on dilational rheology of DPPC–palmitic acid Langmuir monolayers. Soft Matter. 2012;8:3938-48.

[9] Guzmán E, Ferrari M, Santini E, Liggieri L, Ravera F. Effect of silica nanoparticles on the interfacial properties of a canonical lipid mixture. Colloids and Surfaces B: Biointerfaces. 2015;136:971-80.
[10] Kondej D, Sosnowski TR. Interfacial rheology for the assessment of potential health effects of inhaled carbon nanomaterials at variable breathing conditions. Sci Rep. 2020;10:14044.
[11] Schürch S. Surface tension at low lung volumes: Dependence on time and alveolar size. Resp Physiol. 1982;48:339-55.
[12] Wüstneck R, Perez-Gil J, Wüstneck N, Cruz A, Fainerman VB, Pison U. Interfacial properties of pulmonary surfactant layers. Adv Colloid Interface Sci. 2005;117:33-58.
[13] Ravera F, Ferrari M, Santini E, Liggieri L. Influence of surface processes on the dilational visco-elasticity of surfactant solutions. Adv Colloid Interface Sci. 2005;117:75-100.
